# Supplementary material for: The Implication of Low Dose Dimethyl Sulfoxide on Mitochondrial Function and Oxidative Damage in Cultured Cardiac and Cancer Cells
Source: Molecules. 2021 Dec 1;26(23):7305. doi: 10.3390/molecules26237305 (PMC8658933; doi:10.3390/molecules26237305)
Supplement: Supplementary file 1 [file molecules-26-07305-s001.zip › molecules-1458533-supplementary.pdf]

Supplementary

The Implication of Low Dose Dimethyl Sulfoxide on Mitochondrial Function and Oxidative Damage in Cultured Cardiac and Cancer Cells

Nonhlakanipho F. Sangweni [1,2,*], Phiwayinkosi V. Dludla [1], Nireshni Chellan [1,2], Lawrence Mabasa [1], Jyoti Sharma [1] and Rabia Johnson [1,2,*]

1 Biomedical Research and Innovation Platform (BRIP), South African Medical Research Council, Tygerberg 7505, South Africa, nonhlakanipho.sangweni@mrc.ac.za
2 Centre for Cardio-metabolic Research in Africa, Division of Medical Physiology, Faculty of Medicine and Health Sciences, Stellenbosch University, Tygerberg 7505, South Africa.
* Corresponding author: Nonhlakanipho.Sangweni@mrc.ac.za (NFS); rabia.johnson@mrc.ac.za, (RJ)

1. Results

1.1. Cell Viability

An initial cytotoxicity screening was conducted daily from day 1 – day 6 on healthy cardiomyoblasts using an MTT assay. The H9c2 cells were incubated in MTT solution for 1 hr. Here the results demonstrated no significant effect on the DMSO doses ≤ 0.5 % from day 1 to day 5. From day 5, a gradual increase in cellular activity and metabolism was observed in these cells. Interestingly a more significant effect was observed after the sixth of treatment. Based on these findings, we determined that 6 days of treatment would be the suitable treatment duration to study the effects of DMSO (Fig. 1S). From this data, we further data selected four concentrations, the lowest being 0.001% and the highest being 3.7%.

A

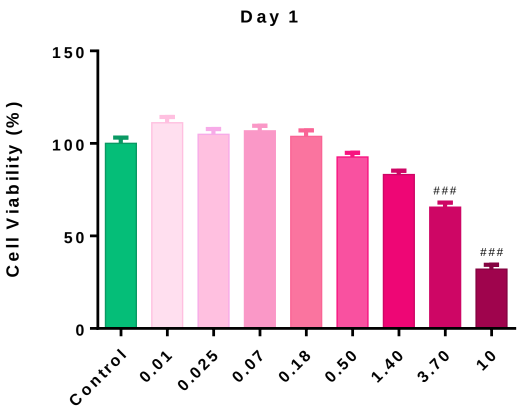

B

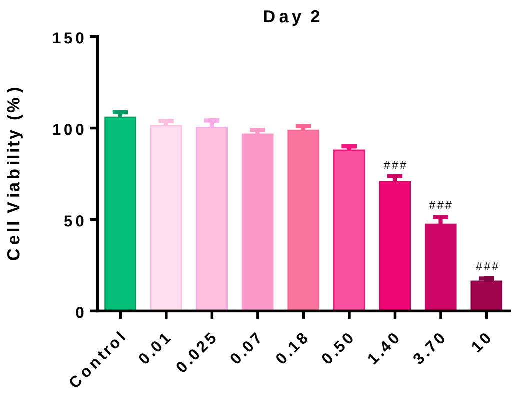

C

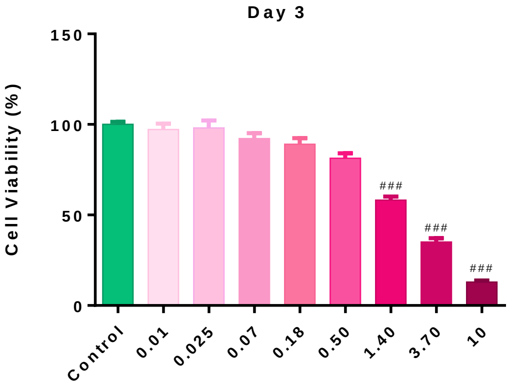

D

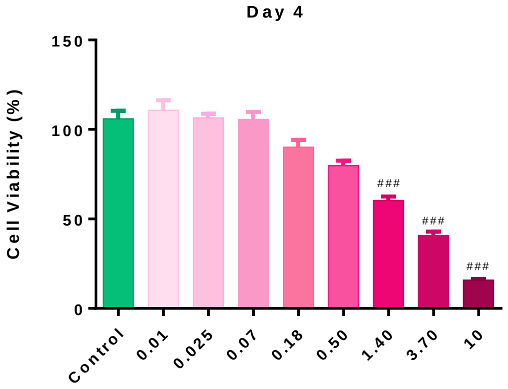

E

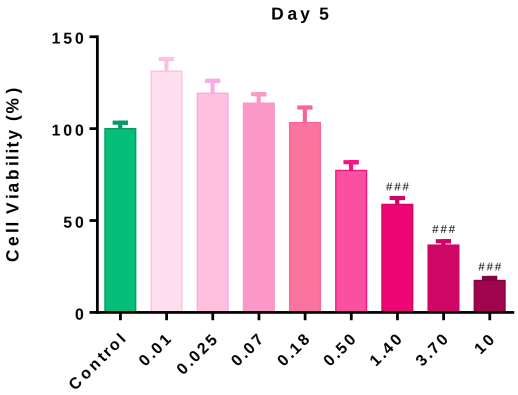

F

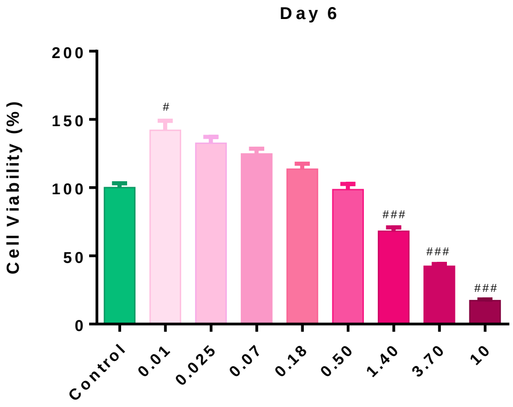

Figure S1. An overview of the cytotoxicity effects of DMSO on the H9c2 cardiomyoblasts. **(A)** Day 1, **(B)** day 2, **(C)** day 3, **(D)** day 4, **(E)** day 5 and**, (F)** day 6. The effect of dimethyl sulfoxide (DMSO) on the cell viability of H9c2 cardiomyoblast and MCF-7 breast cancer cells. Briefly, H9c2 and MCF-7 cells were treated with varying DMSO concentrations (0.001, 0.009, 0.067, 0.5, and 3.7%) every second day for 6 days. Untreated cells served as the control. Data are presented as the mean ± SEM of 3 biological experiments with 5 technical repeats (n = 3). Significance is indicated as $^{\#}p < 0.05$ and $^{\#\#\#}p < 0.001$ versus the control.
